# Synthesis and Biological Activity of 23-Hydroxybetulinic Acid C-28 Ester Derivatives as Antitumor Agent Candidates

**DOI:** 10.3390/molecules17088832

**Published:** 2012-07-25

**Authors:** Yi Bi, Jinyi Xu, Fei Sun, Xiaoming Wu, Wencai Ye, Yijun Sun, Wenwen Huang

**Affiliations:** 1 School of Pharmacy, Yantai University, Yantai 264005, Shandong, China; 2 Department of Medicinal Chemistry, China Pharmaceutical University, Nanjing 210009, Jiangsu, China; 3 State Key Laboratory of Natural Medicines, China Pharmaceutical University, Nanjing 210009, Jiangsu, China; 4 Department of Phytochemistry, China Pharmaceutical University, Nanjing 210009, Jiangsu, China; 5 Drug Screening Center, Nanjing KeyGen Biotech. Co. Ltd., Nanjing 210012, Jiangsu, China

**Keywords:** 23-hydroxybetulinic acid, C-28 ester derivatives, antitumor activity, structure-activity relationships

## Abstract

23-Hydroxybetulinic acid (**1**) served as the precursor for the synthesis of C-28 ester derivatives. The target compounds were evaluated *in vitro* for their antitumor activities against five cell lines (A549, BEL-7402, SF-763, B16 and HL-60)*.* Among the obtained compounds, **6i** had the most potent antitumor activity, with the IC_50_ values of 8.35 µM in HL-60 cells and showed similar antitumor activity as cyclophosphamide in H22 liver tumor and as 5-fluorouracil in B16 melanoma *in vivo*.

## 1. Introduction

**Figure 1:**
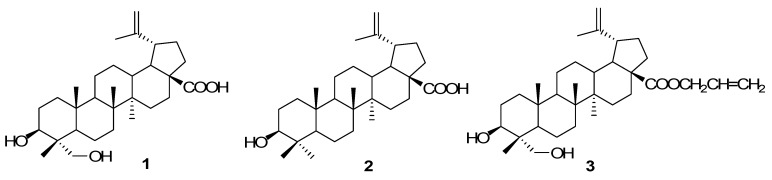
Structure of 23-hydroxybetulinic acid (**1**), betulinic acid (**2**) and a derivative of 23-hydroxybetulinic acid (**3**).

23-Hydroxybetulinic acid (**1**, Figure 1) and betulinic acid (**2**, Figure 1) are well-known members of the class of pentacyclic triterpenes [1,2]. Many biological activities of betulinic acid have been reported, such as antitumor, antiviral, antioxidant and so on [3,4,5,6,7,8,9]. As a good lead compound, betulinic acid showed potent antitumor activity in a series of cell lines and the mechanism of this action might be related to its effects on the proliferation, migration, cell cycle and apoptosis of tumor cells [3,4,5,6,7,8,9].

The chemical structure of 23-hydroxybetulinic acid is similar to that of betulinic acid, and they also have the similar pharmaceutical activities [10]. In our previous study, many derivatives of 23-hydroxy- betulinic acid showed antitumor activity in different cell lines *in vitro* and compound **3** (Figure 1) displayed stronger antitumor activity in mice H22 and B16 than betulinic acid or 23-hydroxybetulinic acid [11]. It was shown that the polarity and length of the chain in C-28 had an important impact on the antitumor activity. These results motivated us to undertake further modifications of the 28-carboxylic acid moiety of lead compound **1**. In this paper, we wish to report a series of 28-carboxylic acid modified 23-hydroxybetulinic acid ester derivatives and their antitumor activity. The preliminary structure-activity relationships are also discussed.

## 2. Results and Discussion

### 2.1. Chemistry

**Scheme 1:**
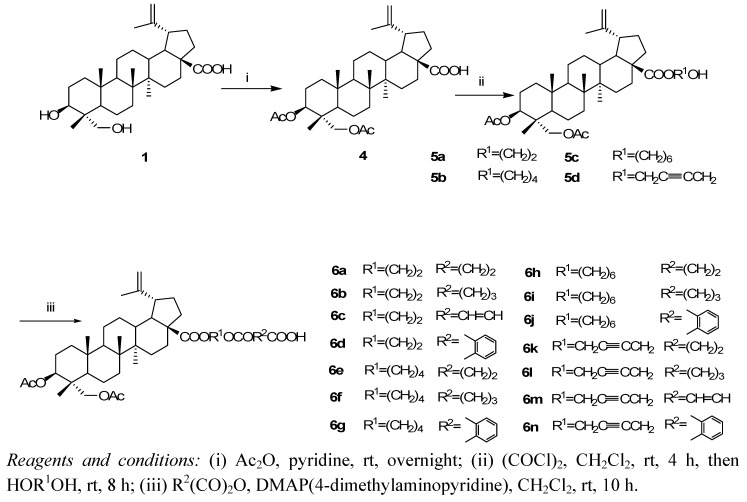
The synthesis of 23-hydroxybetulinic acid C-28 ester derivatives **6a–n**.

For the synthetic experiments, the starting material 23-hydroxybetulinic acid (**1**) was isolated from the root of *Pulsatilla chinensis*. As described in Scheme 1, several derivatives of 23-hydroxybetulinic acid were synthesized. Compounds **5a–d** were readily prepared in two steps starting from compound **1**. Treatment of **5a–d** with anhydrides R_2_(CO)_2_O, such as succinic anhydride, glutaric anhydride, maleic anhydride and phthalic anhydride, in the presence of DMAP in CH_2_Cl_2_ gave compounds **6a–n** in the yields of 53%–76%.

### 2.2. Biological Studies

The MTT (3-(4,5)dimethylthiahiazo (-*z*-y1) -3,5- diphenyltetrazoliumromide) assay results (Table 1) showed that most of the 23-hydroxybetulinic acid derivatives displayed better cytotoxic activities against the tested cells than betulinic acid and 23-hydroxybetulinic acid, especially compounds **6f**, **6h**, and **6i** with IC_50_ values ranging from 8.35 to 28.78 μM. As the assay results showed, C-28 ester derivatives with carboxylic acid substituents at the end of the C-28 side chain and whose C-28 side chain had appropriate length and flexibility exhibited stronger cytotoxicity. The activities of compounds **6k–n** were weaker compared with **6f**, **6h** and **6i**. It was possible due to the alkyne group in the C-28 side chain blocking the bending and rotation of the compounds.

Compound **6i** was chosen to evaluated its antitumor activities in mice *in vivo* based on its significant efficiency *in vitro*. As illustrated in Table 2 and Table 3, in H22 mice, compound **6i** exhibited stronger activity than 23-hydroxybetulinic acid and comparable activity to cyclophosphamide, which was used as a positive control. In the B16 group, compound **6i** also showed more potent activity than 23-hydroxybetulinic acid but somewhat weaker than 5-fluorouracil.

## 3. Experimental 

### 3.1. Synthesis

#### 3.1.1. General

Melting points were obtained on a MEL-TEMP II melting-point apparatus and are uncorrected. IR were recorded on Nicolet Impact 410 or Bruker FT-IR TENSOR27 instruments. ^1^H-NMR spectra were recorded on a Bruker-ACF-300 (chemical shifts are expressed as values relative to TMS as internal standard). HR-MS were obtained using a Agilent QTOF 6520 instrument.

#### 3.1.2. General Procedure for the Synthesis of **6a–n**

Ac_2_O (0.15 mL, 1.5 mmol) was added to a solution of 23-hydroxybetulinic acid (**1**, 120.0 mg, 0.25 mmol) in dry pyridine (5 mL). The mixture was stirred overnight at room temperature. After adding acetic ether (10 mL), the mixture was washed with 9% HCl (20 mL × 3) and brine (20 mL × 3), dried over anhydrous Na_2_SO_4_, filtered, and evaporated to dryness. The residue was purified by crystallization from ethyl acetate to afford the desired compound **4** as yellow powder (136.0 mg, 98%). (COCl)_2_ (0.1 mL) was added to a solution of **4** (100.0 mg, 0.18 mmol) in dry CH_2_Cl_2_ (10 mL). The mixture was stirred for 4 h at room temperature and evaporated to dryness. Immediately, the corresponding alcohol (5 equiv.) and CH_2_Cl_2_ (10 mL) were added and the mixture allowed to stir for 8vh at room temperature, evaporated to dryness and purified by column chromatography on silica gel, eluted with an mixture of petroleum ether/ethyl acetate to give compounds **5a–d**. The corresponding R_2_(CO)_2_O (4 equiv.) was added to a solution of compounds **5a–d** in CH_2_Cl_2_ (10 mL) in the presence of DMAP (1.5 equiv.). The mixture was stirred for 10 h at room temperature and washed with 9% HCl (20 mL × 2), H_2_O (20 mL × 2), brine (20 mL × 2), dried over anhydrous Na_2_SO_4_, filtered, and concentrated to dryness. The residue was purified by column chromatography on silica gel to afford compounds **6a–n**.

*Butanedioic acid, 1-[2-[(3β,23-diacetoxylup-20(29)-en-28-oyl)oxy]ethyl] ester* (**6a**). From 1,2-ethanediol (56.0 mg, 0.9 mmol) and succinic anhydride (55.0 mg, 0.55 mmol); column chromatography with petroleum ether/ethyl acetate/acetic acid = 50:50:1 (v:v:v). Yield: 73.0 mg (76%) as a yellow solid, mp 68 °C–70 °C; IR (KBr, cm^−1^) ν 3429, 2985, 2947, 2872, 1738, 1731, 1649, 1453, 1370, 1246, 1151, 1039, 881; ^1^H-NMR (CDCl_3_, 300 MHz) δ 0.80, 0.88, 0.91, 0.96, 1.68 (3H each, s, 24, 25, 26, 27, 30-CH_3_), 2.02, 2.06 (3H each, s, 3 and 23-OCOCH_3_), 2.67 (4H, m, 28-COOCH_2_CH_2_O COCH_2_CH_2_), 2.99 (1H, m, 19-CH), 3.68 (1H, d, *J =* 11.9 Hz, 23-CH_2_), 3.84 (1H, d, *J =* 11.9 Hz, 23-CH_2_), 4.29 (4H, m, 17-COOCH_2_CH_2_), 4.61 (1H, s, 29=CH_2_), 4.72 (1H, s, 29=CH_2_), 4.74 (1H, m, 3-CH); HR-MS (ESI, M+H) *m/z*: calcd for C_40_H_61_O_10_ 701.4108, found 701.4104.

*Pentanedioic acid, 1-[2-[(3β,23-diacetoxylup-20(29)-en-28-oyl)oxy]ethyl] ester* (**6b**). From 1,2-ethanediol (56.0 mg, 0.9 mmol) and glutaric anhydride (63.0 mg, 0.55 mmol); column chromatography with petroleum ether/ethyl acetate/acetic acid = 50:50:1 (v:v:v). Yield: 71.0 mg (73%) as a yellow oil; IR (KBr, cm^−1^) ν 3457, 2948, 2873, 1738, 1711, 1642, 1451, 1375, 1245, 1153, 1039, 885, 756; ^1^H-NMR (CDCl_3_, 300 MHz) δ 0.80, 0.87, 0.91, 0.96, 1.68 (3H each, s, 24, 25, 26, 27, 30-CH_3_), 2.01, 2.06 (3H each, s, 3 and 23-OCOCH_3_), 2.43 (4H, m, 28-COOCH_2_CH_2_OCOCHCH_2_), 2.98 (1H, m, 19-CH), 3.68 (1H, d, *J* = 11.6 Hz, 23-CH_2_), 3.85 (1H, d, *J* = 11.6 Hz, 23-CH_2_), 4.29 (4H, m, 17-COOCH_2_CH_2_), 4.60 (1H, s, 29=CH_2_), 4.72 (1H, s, 29=CH_2_), 4.75 (1H, m, 3-CH); HR-MS (ESI, M+H) *m/z*: calcd for C_41_H_63_O_10_ 715.4265, found 715.4262.

*Butylenedioic acid, 1-[2-[(3β,23-diacetoxylup-20(29)-en-28-oyl)oxy]ethyl] ester* (**6c**). From 1,2-ethanediol (56.0 mg, 0.9 mmol) and maleic anhydride (54.0 mg, 0.55 mmol); column chromatography with petroleum ether/ethyl acetate = 1:1 (v:v). Yield: 59.0 mg (62%) as a white solid; mp 68 -70 °C; IR (KBr, cm^−1^) ν 3458, 3418, 2945, 2869, 1732, 1640, 1452, 1375, 1245, 1155, 1038, 758; ^1^H-NMR (CDCl_3_, 300 MHz) δ 0.80, 0.87, 0.91, 0.96, 1.68 (3H each, s, 24, 25, 26, 27, 30-CH_3_), 2.01, 2.06 (3H each, s, 3 and 23-OCOCH_3_), 2.95 (1H, m, 19-CH), 3.69(1H, d, *J* = 11.3 Hz, 23-CH_2_), 3.83 (1H, d, *J* = 11.3 Hz, 23-CH_2_), 4.36 (2H, m, 17-COOCH_2_CH_2_), 4.44 (2H, m, 17-COOCH_2_CH_2_), 4.61 (1H, s, 29=CH_2_), 4.72 (1H, s, 29=CH_2_), 4.76 (1H, m, 3-CH), 6.36(1H, d, *J* = 12.8 Hz, 28-COOCH_2_CH_2_OCOCH=CH), 6.50 (1H, d, *J* = 12.8 Hz, 28-COOCH_2_CH_2_OCOCH=CH). HR-MS (ESI, M+H) *m/z*: calcd for C_40_H_59_O_10_ 699.3952, found 699.3959.

*1,2-Xylenedioic acid, 1-[2-[(3β,23-diacetoxylup-20(29)-en-28-oyl)oxy]ethyl] ester* (**6d**). From 1,2-ethanediol (56.0 mg, 0.9 mmol) and phthalic anhydride (81.0 mg, 0.55 mmol); column chromatography with petroleum ether/ethyl acetate/acetic acid = 50:50:1 (v:v:v). Yield: 75.0 mg (73%) as a white solid, mp 70 °C–72 °C; IR (KBr, cm^−1^) ν 3450, 2947, 2871, 1731, 1635, 1448, 1380, 1248, 1128, 1038, 883, 743; ^1^H-NMR (CDCl_3_, 300 MHz) δ 0.79, 0.82, 0.89, 0.92, 1.65 (3H each, s, 24, 25, 26, 27, 30-CH_3_), 2.01, 2.06 (3H each, s, 3 and 23-OCOCH_3_), 2.97 (1H, m, 19-CH), 3.68 (1H, d, *J* = 11.6 Hz, 23-CH_2_), 3.83 (1H, d, *J* = 11.6 Hz, 23-CH_2_), 4.58 (7H, m, 29 = CH2, 3-CH and 17-COOCH_2_CH_2_), 7.70 (4H, m, 17-COOCH_2_CH_2_OCOC_6_H_4_COOH); HR-MS (ESI, M+H) *m/z*: calcd for C_44_H_61_O_10_ 749.4108, found 749.4111.

*Butanedioic acid, 1-[4-[(3β,23-diacetoxylup-20(29)-en-28-oyl)oxy]ethyl] ester* (**6e**). From 1,4-butanediol (81.0 mg, 0.9 mmol) and succinic anhydride (44.0 mg, 0.44 mmol); column chromatography with petroleum ether/ethyl acetate/acetic acid = 50:50:1 (v:v:v). Yield: 56.0 mg (70%) as a yellow oil; IR (KBr, cm^−1^) ν 3488, 3422, 2923, 2854, 1738, 1641, 1461, 1377, 1245, 1157, 1037, 974, 883; ^1^H-NMR (CDCl_3_, 300 MHz) δ 0.80, 0.88, 0.91, 0.96, 1.68 (3H each, s, 24, 25, 26, 27, 30-CH_3_), 2.01, 2.06 (3H each, s, 3 and 23-OCOCH_3_), 2.65 (4H, m, 28-COOCH_2_(CH_2_)_2_CH_2_OCOCH_2_CH_2_), 2.99 (1H, m, 19-CH), 3.68 (1H, d, *J* = 11.7 Hz, 23-CH_2_), 3.84 (1H, d, *J* = 11.7 Hz, 23-CH_2_), 4.13 (4H, m, 17-COOCH_2_(CH_2_)_2_CH_2_), 4.60 (1H, s, 29=CH_2_), 4.72 (1H, s, 29=CH_2_), 4.74 (1H, m, 3-CH); HR-MS (ESI, M+H) *m/z*: calcd for C_42_H_65_O_10_ 729.4421, found 729.4428.

*Pentanedioic acid, 1-[4-[(3β,23-diacetoxylup-20(29)-en-28-oyl)oxy]ethyl] ester* (**6f**). From 1,4-butanediol (81.0 mg, 0.9 mmol) and glutaric anhydride (50.0 mg, 0.44 mmol); column chromatography with petroleum ether/ethyl acetate/acetic acid = 50:50:1 (v:v:v). Yield: 55.0 mg (67%) as a yellow oil; IR (KBr, cm^−1^) ν 3425, 2947, 2871, 1735, 1641, 1451, 1384, 1244, 1155, 1036, 975; ^1^H-NMR (CDCl_3_, 300 MHz) δ 0.80, 0.87, 0.91, 0.96, 1.70 (3H each, s, 24, 25, 26, 27, 30-CH_3_), 2.01, 2.06 (3H each, s, 3 and 23-OCOCH_3_), 2.43 (4H, m, 28-COOCH_2_(CH_2_)_2_CH_2_OCOCH_2_CH_2_CH_2_), 2.99 (1H, m, 19-CH), 3.68 (1H, d, *J* = 12.3 Hz, 23-CH_2_), 3.85 (1H, d, *J* = 12.3 Hz, 23-CH_2_), 4.10 (4H, m, 28-COOCH_2_(CH_2_)_2_CH_2_), 4.60 (1H, s, 29=CH_2_), 4.73 (1H, s, 29=CH_2_), 4.76 (1H, m, 3-CH); HR-MS (ESI, M+H) *m/z*: calcd for C_43_H_67_O_10_ 743.4578, found 743.4573.

*1,2-xylenedioic acid, 1-[4-[(3β,23-diacetoxylup-20(29)-en-28-oyl)oxy]ethyl] ester* (**6g**). From 1,4-butanediol (81.0 mg, 0.9 mmol) and phthalic anhydride (65.0 mg, 0.44 mmol); column chromatography with petroleum ether/ethyl acetate/acetic acid = 50:50:1 (v:v:v). Yield: 53.0 mg (62%) as a white solid, mp 163 °C–165 °C; IR (KBr, cm^−1^) ν 3457, 2973, 2866, 1643, 1455, 1381, 1055, 1025, 1012, 771; ^1^H-NMR (CDCl_3_, 300 MHz) δ 0.80, 0.88, 0.91, 0.95, 1.68 (3H each, s, 24, 25, 26, 27, 30-CH_3_), 2.02, 2.06 (3H each, s, 3 and 23-OCOCH_3_), 2.99 (1H, m, 19-CH), 3.69 (1H, d, *J* = 11.6 Hz, 23-CH_2_), 3.84 (1H, d, *J* = 11.6 Hz, 23-CH_2_), 4.12 (4H, m, 28-COOCH_2_(CH_2_)_2_CH_2_), 4.60 (1H, s, 29=CH_2_), 4.72 (1H, s, 29 = CH_2_), 4.76 (1H, m, 3-CH), 7.52 (4H, m, 17-COO(CH_2_)_4_OCOC_6_H_4_COOH); HR-MS (ESI, M+H) *m/z*: calcd for C_46_H_65_O_10_ 777.4421, found 777.4427.

*Butanedioic acid, 1-[6-[(3β,23-diacetoxylup-20(29)-en-28-oyl)oxy]ethyl] ester* (**6h**). From 1,6-hexanediol (106.0 mg, 0.9 mmol) and succinic anhydride (42.0 mg, 0.42 mmol); column chromatography with petroleum ether/ethyl acetate = 2:1 (v:v). Yield: 58.0 mg (73%) as a yellow oil; IR (KBr, cm^−1^) ν 3480, 3417, 2945, 2867, 1738, 1641, 1454, 1372, 1244, 1160, 1038, 885, 756; ^1^H-NMR (CDCl_3_, 300 MHz) δ 0.80, 0.88, 0.91, 0.96, 1.68 (3H each, s, 24, 25, 26, 27, 30-CH_3_), 2.01, 2.06 (3H each, s, 3 and 23-OCOCH_3_), 2.65 (4H, m, 17-COOCH_2_(CH_2_)_4_CH_2_OCOCH_2_CH_2_), 2.99 (1H, m, 19-CH), 3.68 (1H, d, *J* = 11.5 Hz, 23-CH_2_), 3.86 (1H, d, *J* = 11.5 Hz, 23-CH_2_), 4.05 (4H, m, 17-COOCH_2_(CH_2_)_4_CH_2_), 4.60 (1H, s, 29=CH_2_), 4.72 (1H, s, 29=CH_2_), 4.74 (1H, m, 3-CH); HR-MS (ESI, M+H) *m/z*: calcd for C_44_H_69_O_10_ 757.4734, found 757.4738.

*Pentanedioic acid, 1-[6-[(3β,23-diacetoxylup-20(29)-en-28-oyl)oxy]ethyl] ester* (**6i**). From 1,6-hexanediol (106 mg, 0.9 mmol) and glutaric anhydride (48.0 mg, 0.42 mmol); column chromatography with petroleum ether/ethyl acetate = 1:2 (v:v). Yield: 55.0 mg (68%) as a yellow oil; IR (KBr, cm^−1^) ν 3454, 2943, 2866, 1731, 1642, 1454, 1378, 1247, 1157, 1054, 771; ^1^H-NMR (CDCl_3_, 300 MHz) δ 0.80, 0.87, 0.91, 0.96, 1.68 (3H each, s, 24, 25, 26, 27, 30-CH_3_), 1.98, 2.06 (3H each, s, 3 and 23-OCOCH_3_), 2.41 (4H, m, 28-COOCH_2_(CH_2_)_4_CH_2_OCOCH_2_CH_2_CH_2_), 2.99 (1H, m, 19-CH), 3.69 (1H, d, *J* = 10.2 Hz, 23-CH_2_), 3.83 (1H, d, *J* = 10.2 Hz, 23-CH_2_), 4.06 (4H, m, 28-COOCH_2_(CH_2_)_4_ CH_2_), 4.60 (1H, s, 29=CH_2_), 4.72 (1H, s, 29=CH_2_), 4.74 (1H, m, 3-CH); HR-MS (ESI, M+H) *m/z*: calcd for C_45_H_71_O_10_ 771.4891, found 771.4896.

*1*,*2-Xylenedioic acid, 1-[6-[(3β,23-diacetoxylup-20(29)-en-28-oyl)oxy]ethyl] ester* (**6j**). From 1,6-hexanediol (106.0 mg, 0.9 mmol) and phthalic anhydride (62.0 mg, 0.42 mmol); column chromatography with petroleum ether/ethyl acetate/acetic acid = 50:50:1 (v:v:v). Yield: 50.0 mg (59%) as a yellow oil; IR (KBr, cm^−1^) ν 3479, 2946, 2866, 1735, 1704, 1591, 1384, 1369, 1246, 1130, 1040, 652; ^1^H-NMR (CDCl_3_, 300 MHz) δ 0.79, 0.87, 0.91, 0.95, 1.68 (3H each, s, 24, 25, 26, 27, 30-CH_3_), 2.01, 2.05 (3H each, s, 3 and 23-OCOCH_3_), 2.99 (1H, m, 19-CH), 3.68(1H, d, *J* = 11.6 Hz, 23-CH_2_), 3.84 (1H, d, *J* = 11.6 Hz, 23-CH_2_), 4.07 (4H, m, 28-COOCH_2_(CH_2_)_4_CH_2_), 4.60 (1H, s, 29=CH_2_), 4.73 (1H, s, 29=CH_2_), 4.76 (1H, m, 3-CH), 7.48 (4H, m, 17-COO(CH_2_)_6_OCOC_6_H_4_COOH); HR-MS (ESI, M+H) *m/z*: calcd for C_48_H_69_O_10_ 805.4734, found 805.4739.

*Butanedioic acid, 1-[4-[(3β,23-diacetoxylup-20(29)-en-28-oyl)oxy]butynyl] ester* (**6k**). Butyne-1,4-diol (77.0 mg, 0.9 mmol) and succinic anhydride (37.0 mg, 0.37 mmol); column chromatography with petroleum ether/ethyl acetate/acetic acid = 50:50:1 (v:v:v). Yield: 50.0 mg (64%) as a yellow solid, mp 99 °C–101 °C; IR (KBr, cm^−1^) ν 3450, 2929, 2870, 1736, 1649, 1427, 1370, 1246, 1148, 1041, 971, 803, 641; ^1^H-NMR (CDCl_3_, 300 MHz) δ 0.80, 0.87, 0.90, 0.96, 1.68(3H each, s, 24, 25, 26, 27, 30-CH_3_), 2.01, 2.06 (3H each, s, 3 and 23-OCOCH_3_), 2.69 (4H, t, 17- 17-COOCH_2_C≡CCH_2_O COCH_2_CH_2_COOH), 2.99 (1H, m, 19-CH), 3.68 (1H, d, *J* = 11.7 Hz, 23-CH2), 3.85 (1H, d, *J* = 11.7 Hz, 23-CH2), 4.69 (7H, m, 29=CH_2_, 3-CH and 17-COOCH_2_C≡CCH_2_); HR-MS (ESI, M+H) *m/z*: calcd for C_42_H_61_O_10_ 725.4108, found 725.4102.

*Pentanedioic acid, 1-[4-[(3β,23-diacetoxylup-20(29)-en-28-oyl)oxy]butynyl] ester* (**6l**). From butyne-1,4-diol (77.0 mg, 0.9 mmol) and glutaric anhydride (42.0 mg, 0.37 mmo); column chromatography with petroleum ether/ethyl acetate/acetic acid = 50:50:1 (v:v:v). Yield: 50.0 mg (67%) as a yellow solid, mp 90 °C–92 °C; IR (KBr, cm^−1^) ν 3438, 2946, 2871, 1737, 1643, 1582, 1451, 1369, 1245, 1146, 1123, 1041, 973; ^1^H-NMR (DMSO, 300 MHz) δ 0.76, 0.83, 0.87, 0.94, 1.65 (3H each, s, 24, 25, 26, 27, 30-CH_3_), 1.96, 1.99 (3H each, s, 3 and 23-OCOCH_3_), 2.19 (4H, m, 17-COOCH_2_ C≡CCH_2_OCOCH_2_CH_2_CH_2_COOH), 2.91 (1H, m, 19-CH), 3.63 (1H, d, *J* = 11.9 Hz, 23-CH_2_), 3.78 (1H, d, *J* = 11.9 Hz, 23-CH_2_), 4.70 (7H, m, 29=CH_2_, 3-CH and 17-COOCH_2_≡CCH_2_); HR-MS (ESI, M+H) *m/z*: calcd for C_43_H_63_O_10_ 739.4265, found 739.4261.

*Butylenedioic acid, 1-[4-[(3β,23-diacetoxylup-20(29)-en-28-oyl)oxy]butynyl] ester* (**6m**). From butyne-1,4-diol (77.0 mg, 0.9 mmol) and maleic anhydride (36.0 mg, 0.37 mmol); column chromatography with petroleum ether/ethyl acetate = 1:2 (v:v). Yield: 36.0 mg (53%) as a yellow oil; IR (KBr, cm^−1^) ν 3405, 2967, 2922, 2872, 1731, 1650, 1584, 1437, 1407, 1334, 1126, 1044, 953; ^1^H-NMR (DMSO, 300 MHz) δ 0.76, 0.82, 0.87, 0.94, 1.65 (3H each, s, 24, 25, 26, 27, 30-CH_3_), 1.96, 1.99 (3H each, s, 3 and 23-OCOCH_3_), 3.39 (1H, m, 19-CH), 3.63 (1H, d, *J* = 11.4 Hz, 23-CH_2_), 3.78 (1H, d, *J* = 11.4 Hz, 23-CH_2_), 4.69 (7H, m, 29=CH_2_, 3-CH and 17-COOCH_2_C≡CCH_2_), 6.22 (1H, d, *J =* 15.8 Hz, 17-COOCH_2_C≡CCH_2_OCOCH=CHCOOH), 6.77 (1H, d, *J =* 15.8 Hz, 17-COOCH_2_C≡CCH_2_OCO CH=CHCOOH); HR-MS (ESI, M+H) *m/z*: calcd for C_42_H_59_O_10_ 723.3952, found 723.3948.

*1,2-Xylenedioic acid, 1-[4-[(3β,23-diacetoxylup-20(29)-en-28-oyl)oxy]butynyl] ester* (**6n**). From butyne-1,4-diol (77.0 mg, 0.9 mmol) and phthalic anhydride (55.0 mg, 0.37 mmol); column chromatography with petroleum ether/ethyl acetate/acetic acid = 50:50:0.4 (v:v:v). Yield: 44.0 mg (61%) as a yellow oil; IR (KBr, cm^−1^) ν 3435, 3070, 2948, 2871, 1728, 1591, 1450, 1385, 1247, 1131, 1040, 747; ^1^H-NMR (CDCl_3_, 300 MHz) δ 0.82, 0.88, 0.91, 0.98, 1.71 (3H each, s, 24, 25, 26, 27, 30-CH_3_), 2.03, 2.08 (3H each, s, 3 and 23-OCOCH_3_), 3.06 (1H, m, 19-CH), 3.71 (1H, d, *J* = 11.7 Hz, 23-CH_2_), 3.86 (1H, d, *J* = 11.7 Hz, 23-CH_2_), 4.83 (7H, m, 29=CH_2_, 3-CH and 17-COOCH_2_C≡CCH_2_), 7.78 (4H, m, 17-COOCH_2_C≡CCH_2_OCOC_6_H_4_COOH); HR-MS (ESI, M+H) *m/z*: calcd for C_46_H_61_O_10_ 773.4108, found 773.4101.

### 3.2. Pharmacology

*In vitro*, the cytotoxic activities of 23-hydroxybetulinic acid, betulinic acid and all derivatives were determined by the MTT cytotoxicity assay, which was performed in 96-well plates. The tumor cell line panel consisted of A549 (human lung carcinoma), BEL-7402 (human hepatoma), SF-763 (human cerebroma), B16 (mice melanoma), HL-60 (human leukaemia) (final concentration in the growth medium was 2~4 × 10^4^ /mL). MTT solution (20 μL/well) was added after cells were treated with drug for 48 h, and cells were incubated for a further 4 h at 37 °C. The purple formazan crystals were dissolved in 150 μL DMSO. After 5 min, the plates were read on an automated microplate spectrophotometer at 570 nm. Assays were performed in triplicate in three independent experiments. The concentration required for 50% inhibition of cell viability (IC_50_) what was calculated. In all of these experiments, three replicate wells were used to determine each point [11].

*In vivo*, ICR female mice with body weight of 18–22 g were transplanted with H22 and B16 subcutaneously into the right axilla according to protocols of transplant tumor research. After 24 h of tumor transplantation, mice were weighed, and each model group was at random divided into 4 groups, each of which had 10 mice in H22 and B16 group. The groups with 23-OH betulinic acid and **6i** were administered intraperitoneously 25 mg/kg in a vehicle of 20% DMSO/80% saline. respectively. The positive control group was treated with cyclophosphamide (30 mg/kg) in H22 group and 5-fluorouracil (25 mg/kg) in B16 group through intravenous injection in a vehicle of 20% DMSO/80% saline. The negative control group received 0.9% normal saline through intravenous injection. All test compounds were given through injections 24 h after tumor transplantation (or inoculation). Treatments were done at a frequency of intravenous or intraperitoneal injection one dose per day for a total of four consecutive days in H22 group and for a total of 11 consecutive days in B16 group. After the treatments, all mice were killed and weighed simultaneously, and then segregated and weighed the tumor [11]. Tumor inhibitory ratio was calculated by the following formula and perform T test:

Tumor inhibitory ratio (%) = (1-average tumor weight of treated group/average tumor weight of control group) × 100%

## 4. Conclusions

In summary, a series of novel of 23-hydroxybetulinic acid C-28 ester derivatives were synthesized and tested for their *in vitro* cytotoxic activities against five human tumor cell lines. Most of the compounds showed moderate potent cytotoxic activities on all the tested cells. The results of preliminary biological activity showed that three compounds (**6f**, **6h**, **6i**) possessed impressive cytotoxicities. Compound **6i** was chosen to evaluate its antitumor activities *in vivo*. Compound **6i** showed similar antitumor activity as cyclophosphamide in H22 and as 5-fluorouracil in B16. Further structure modification and SAR studies of antitumor 23-hydroxybetulinic acid derivatives are in progress in our laboratory and the results will be reported in due course [12].

## Figures and Tables

**Table 1:** The cytotoxicity data of 23-hydroxybetulinic acid and its derivatives [IC_50_ (μmol/L) ± SD].

Compound	Cell line				
	A549	BEL-7402	SF-763	B16	HL-60
**HBA**	81.36 ± 3.54	89.81 ± 7.32	90.09 ± 8.31	75.64 ± 8.55	80.54 ± 9.13
**BA**	89.62 ± 11.23	52.51 ± 2.55	78.89 ± 9.24	50.09 ± 7.32	76.77 ± 10.58
**6a**	34.12 ± 11.10	43.06 ± 9.71	42.45 ± 12.11	31.04 ± 9.48	22.85 ± 9.04
**6b**	35.18 ± 15.42	48.90 ± 11.56	51.05 ± 9.75	46.06 ± 1.56	35.37 ± 14.35
**6c**	27.12 ± 9.84	36.81 ± 12.05	43.67 ± 15.70	32.15 ± 4.02	28.63 ± 9.31
**6d**	68.48 ± 13.10	59.37 ± 9.57	63.51 ± 8.92	51.57 ± 8.10	49.18 ± 10.48
**6e**	31.36 ± 19.04	22.06 ± 11.75	34.04 ± 9.11	29.12 ± 7.27	14.80 ± 7.91
**6f**	12.35 ± 1.80	17.08 ± 5.09	19.52 ± 9.01	15.22 ± 8.67	12.94 ± 6.55
**6g**	69.82 ± 8.14	57.81 ± 15.85	73.10 ± 15.02	72.13 ± 2.52	52.73 ± 10.24
**6h**	15.80 ± 6.51	28.78 ± 9.12	21.84 ± 3.41	19.09 ± 6.42	10.03 ± 1.76
**6i**	10.02 ± 5.04	11.15 ± 9.34	12.32 ± 6.10	14.05 ± 2.97	8.35 ± 4.28
**6j**	62.72 ± 5.03	59.25 ± 10.84	49.61 ± 12.46	40.43 ± 10.06	39.66 ± 9.75
**6k**	57.09 ± 16.13	69.09 ± 7.55	53.74 ± 9.82	43.48 ± 9.77	37.09 ± 14.63
**6l**	40.86 ± 8.44	55.62 ± 9.02	42.88 ± 10.39	35.70 ± 15.12	30.53 ± 8.34
**6m**	51.71 ± 10.05	68.13 ± 5.89	55.43 ± 13.74	46.56 ± 11.84	47.61 ± 2.45
**6n**	60.16 ± 9.58	76.92 ± 12.70	66.75 ± 16.83	50.40 ± 15.22	56.23 ± 14.91

BA: betulinic acid, HBA: 23-hydroxybetulic acid; data is mean of three experiments.

**Table 2:** The cytoxicity data of 23-hydroxybetulinic acid and its derivative **6i** in mice H22 *in vivo*.

Drugs	Dose	Injection	Number of mice		Weight of mice (g)		Weight of tumor X ± SD (g)	Ratio of inhibition (%)	*P* value
			Start	End	Start	End			
**Normal saline**	0.4 mL/mouse	iv	10	10	18.8 ± 1.1	25.3 ± 1.2	1.70 ± 0.45		
**CP**	30 mg/kg	iv	10	10	18.4 ± 1.2	25.2 ± 1.6	0.71 ± 0.27	58.2	<0.01
**HBA**	25 mg/kg	ip	10	10	18.5 ± 1.6	26.1 ± 1.5	1.31 ± 0.75	22.9	>0.05
**6i**	25 mg/kg	ip	10	10	18.3 ± 0.9	25.7 ± 1.4	0.75 ± 0.63	55.9	<0.01

CP – cyclophosphamide; HBA – 23-hydroxybetulinic acid.

**Table 3:** The cytoxicity data of 23-hydroxybetulinic acid and its derivative **6i** in mice B16 *in vivo*.

Drugs	Dose	Injection	Number of mice		Weight of mice (g)		Weight of tumor X ± SD (g)	Ratio of inhibition (%)	*P* value
			Start	End	Start	End			
**Normal Saline**	0.4 mL/mouse	iv	10	10	18.9 ± 2.1	23.8 ± 1.6	1.96 ± 0.52		
**5-FU**	25 mg/kg	iv	10	10	19.4 ± 1.8	23.2 ± 1.8	0.59 ± 0.31	69.9	<0.01
**HBA**	25 mg/kg	ip	10	10	19.1 ± 1.7	24.2 ± 1.7	1.59 ± 0.44	18.9	>0.05
**6i**	25 mg/kg	ip	10	10	18.9 ± 1.4	24.7 ± 1.1	0.81 ± 0.21	58.7	<0.01

5-FU – 5-fluorouracil; HBA – 23-hydroxybetulinic acid.
